# Monitoramento da Temperatura Esofágica na Ablação da Fibrilação Atrial: Evidências em Evolução e Novas Perspectivas

**DOI:** 10.36660/abc.20250471

**Published:** 2025-09-05

**Authors:** Silvia Helena Cardoso Boghossian

**Affiliations:** 1 Hospital Universitário Pedro Ernesto Rio de Janeiro RJ Brasil Hospital Universitário Pedro Ernesto, Rio de Janeiro, RJ – Brasil; 2 Complexo Hospitalar Américas – Hospital Vitória e Hospital Samaritano Barra Rio de Janeiro RJ Brasil Complexo Hospitalar Américas – Hospital Vitória e Hospital Samaritano Barra, Rio de Janeiro, RJ – Brasil

**Keywords:** Fibrilação Atrial, Ablação por Radiofrequência, Temperatura, Esôfago

A ablação por radiofrequência (RF) da fibrilação atrial (FA) é uma estratégia terapêutica amplamente utilizada, com crescente eficácia e sofisticação técnica. Entretanto, o procedimento não é isento de riscos, sendo a fístula átrio-esofágica uma complicação rara, porém de extrema gravidade. Nesse contexto, o monitoramento da temperatura esofágica (luminal esophageal temperature – LET) surgiu como uma medida de proteção adicional, embora sua eficácia tenha sido objeto de controvérsia na literatura.

O estudo "Monitoramento da Temperatura Esofágica durante a Ablação de Fibrilação Atrial: Um Estudo Randomizado"^1^ apresenta evidências relevantes e oportunas sobre o impacto clínico do LET, especialmente ao comparar sondas de sensor único com sondas multissensoriais durante a ablação de FA. Trata-se de uma abordagem metodológica robusta, com desenho randomizado, que contribui significativamente para o refinamento das práticas de segurança no procedimento.

Diversos estudos prévios demonstraram resultados divergentes quanto à utilidade do LET.^2^ Singh et al.^3^ relataram redução significativa na incidência de lesões esofágicas com o uso de sondas térmicas. Por outro lado, o estudo OPERA, de caráter multicêntrico e randomizado, não evidenciou benefício claro na utilização do LET em termos de redução de lesões detectadas por endoscopia.^4^ Além disso, trabalhos como o de Ayoub et al.^5^ mostraram que picos de temperatura esofágica não necessariamente se correlacionam com a presença de lesões mucosas ou submucosas. Tais dados levaram a questionamentos quanto à sensibilidade e especificidade do monitoramento térmico esofágico tradicional.

Neste cenário, o estudo publicado no ABC Cardiol^1^ se destaca por oferecer uma comparação direta entre sondas de sensor único e sondas multissensoriais. Os autores demonstraram, de forma consistente, a superioridade da sonda multissensor na detecção de aumentos críticos de temperatura esofágica, permitindo intervenções clínicas imediatas — como a interrupção da energia ou o ajuste de potência — e resultando em menor incidência de lesões esofágicas na endoscopia de seguimento.

Tal benefício pode ser atribuído à maior cobertura longitudinal e à distribuição térmica mais abrangente proporcionada pela sonda multissensor, que monitora simultaneamente diferentes pontos ao longo do trajeto esofágico. Estudos anteriores, como o de Carroll et al.,^6^ já haviam sugerido essa vantagem técnica, demonstrando maior sensibilidade na detecção de episódios de hipertermia com as sondas multissensoriais em comparação às de sensor único.

Adicionalmente, os resultados apontam para menor incidência de sintomas gastrointestinais no pós-procedimento nos pacientes do grupo multissensor, sugerindo não apenas eficácia preventiva, mas também impacto positivo na recuperação clínica. Halbfass et al.^7^ corroboram esses achados ao demonstrar, em estudo controlado, redução da lesão esofágica com sondas multissensoriais dotadas de múltiplos termopares.

Apesar dessas evidências favoráveis, é importante reconhecer que o monitoramento térmico — mesmo com sondas avançadas — não elimina completamente o risco de lesão. A distância entre o esôfago e a parede posterior do átrio esquerdo, bem como variações anatômicas e técnicas, ainda representam desafios para a detecção precisa e a prevenção total da lesão térmica. Entretanto, a adoção de sondas com múltiplos sensores representa uma evolução significativa na estratégia de proteção esofágica, sendo, atualmente, a abordagem mais sensível e reativa disponível.^8^

Dessa forma, os resultados apresentados neste estudo devem influenciar as práticas clínicas e as futuras diretrizes de segurança na ablação da FA. A implementação rotineira do monitoramento com sondas multissensoriais, particularmente em centros com protocolos de alta potência, pode representar uma medida eficaz para aumentar a segurança do procedimento sem comprometer sua eficácia.
