# Supplementary material for: Monitoramento da Temperatura Esofágica na Ablação da Fibrilação Atrial: Evidências em Evolução e Novas Perspectivas
Source: Arq Bras Cardiol. 2025 Sep 5;122(7):e20250471. [Article in Portuguese] doi: 10.36660/abc.20250471 (PMC12695103; doi:10.36660/abc.20250471)
Supplement: Translation

# Translation: Esophageal Temperature Monitoring during Atrial Fibrillation Ablation: Evolving Evidence and New Perspectives

**DOI:** 10.36660/abc.20250471i

**Authors:** Silvia Helena Cardoso Boghossian

**Affiliations:** 1 Rio de Janeiro RJ Brazil Hospital Universitário Pedro Ernesto, Rio de Janeiro, RJ – Brazil; 2 Rio de Janeiro RJ Brazil Complexo Hospitalar Américas – Hospital Vitória e Hospital Samaritano Barra, Rio de Janeiro, RJ – Brazil

**Keywords:** Atrial Fibrillation, Radiofrequency Ablation, Temperature, Esophagus

Radiofrequency (RF) ablation of atrial fibrillation (AF) is a widely used therapeutic strategy, with increasing efficacy and technical sophistication. However, the procedure is not without risks, with atrioesophageal fistula being a rare but extremely serious complication. In this context, luminal esophageal temperature (LET) monitoring has emerged as an additional protective measure, although its effectiveness has been the subject of controversy in the literature.

The study "Esophageal Temperature Monitoring during Atrial Fibrillation Ablation: A Randomized Study"^1^presents relevant and timely evidence regarding the clinical impact of LET, especially in comparing single-sensor versus multi-sensor probes during AF ablation. It is a methodologically robust, randomized study that significantly contributes to refining safety practices in this procedure.

Previous studies have reported divergent results regarding the utility of LET.^2^Singh et al.^3^observed a significant reduction in the incidence of esophageal lesions with the use of thermal probes. On the other hand, the OPERA trial, a multicenter randomized study, did not show a clear benefit of LET use in reducing endoscopically detected lesions.^4^Additionally, studies such as Ayoub et al.^5^showed that esophageal temperature spikes do not necessarily correlate with mucosal or submucosal injuries. These findings raised concerns about the sensitivity and specificity of traditional esophageal thermal monitoring.

In this scenario, the study published in ABC Cardiol^1^stands out by providing a direct comparison between single-sensor and multi-sensor probes. The authors consistently demonstrated the superiority of the multi-sensor probe in detecting critical esophageal temperature rises, allowing immediate clinical interventions—such as energy interruption or power adjustment—and resulting in a lower incidence of esophageal lesions on follow-up endoscopy.

This benefit may be attributed to the greater longitudinal coverage and broader thermal distribution provided by the multi-sensor probe, which simultaneously monitors different points along the esophageal path. Previous studies, such as Carroll et al.,^6^have already suggested this technical advantage, showing increased sensitivity in detecting hyperthermia episodes with multi-sensor probes compared to single-sensor ones.

Additionally, the results point to a lower incidence of gastrointestinal symptoms in the post-procedure period among patients in the multi-sensor group, suggesting not only preventive efficacy but also a positive impact on clinical recovery. Halbfass et al.^7^support these findings by demonstrating, in a controlled study, a reduction in esophageal injury with multi-sensor probes equipped with multiple thermocouples.

Despite this favorable evidence, it is important to recognize that thermal monitoring—even with advanced probes—does not completely eliminate the risk of injury. The distance between the esophagus and the posterior wall of the left atrium, along with anatomical and technical variations, still pose challenges to accurate detection and full prevention of thermal injury. However, the adoption of multi-sensor probes represents a significant advancement in esophageal protection strategies and currently stands as the most sensitive and responsive approach available.^8^

Therefore, the results presented in this study should influence clinical practice and future safety guidelines in AF ablation. Routine implementation of multi-sensor probe monitoring, particularly in centers with high-power protocols, may represent an effective measure to enhance procedural safety without compromising efficacy.
